# Supplementary material for: An Adult Mouse Model of Dilated Cardiomyopathy Caused by Inducible Cardiac-Specific Bis Deletion
Source: Int J Mol Sci. 2021 Jan 29;22(3):1343. doi: 10.3390/ijms22031343 (PMC7866246; doi:10.3390/ijms22031343)
Supplement: Supplementary file 1 [file ijms-22-01343-s001.pdf]

Table S1. Primary antibodies used in this study.

Target Protein	Provider	Dilution
BIS	Ref (1)	1:10000
HSPB8	Abcam (*Cambridge, U.K.)*	1:500
HSP70	Enzolife Sciences (Farmingdale, NY, USA)	1:500
DESMIN	Abcam	1:100000
GAPDH	Santacruz (*Dallas, TX, USA)*	1:500

Table S2. Primer sequences for qRT-PCR in this study.

Gene	Forward (5'→3')	Reverse (5'→3')
BIS	ACTCTAAGCCTGTTTCCCAGAAGT	AGACTTGTACTTGACCTGGGACAT
ANF	GATAGATGAAGGCAGGAAGCCGC	AGGATTGGAGCCCAGAGTGGACTAGG
BNP	TGTTTCTGCTTTTCCTTTATCTGTC	CTCCGACTTTTCTCTTATCAGCTC
β MHC	ATGTGCCGGACCTTGGAA	CCTCGGGTTAGCTGAGAGATCA
Col1a1	TCACCAAACTCAGAAGATGTAGGA	GACCAGGAGGACCAGGAAG
Col3a1	ACAGCAGTCCAACGTAGATGAAT	TCACAGATTATGTCATCGCAAAG
18s	GGAAGGGCACCACCAGGAGT	TGCAGCCCCGGACATCTAAG

Supplementary figures and figure legends

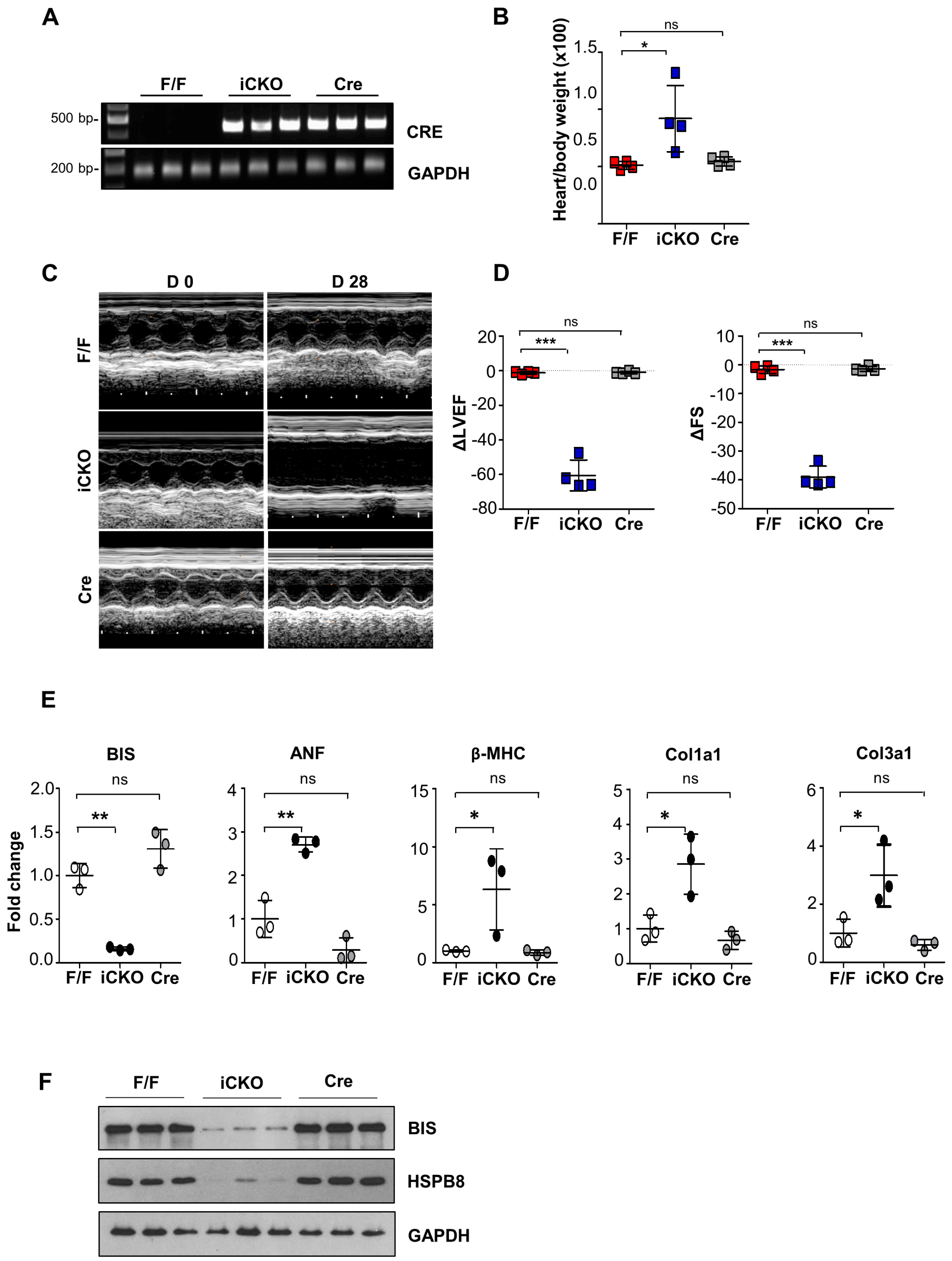

Figure S1. Effect of Cre expression alone on cardiac function.

To investigate the individual effect of Cre expression on cardiac phenotypes, α-MHC-MerCreMer transgenic (Cre) mice 12–16 weeks of age were treated with tamoxifen, as shown in Fig. 1. The cardiac function of Cre mice was compared with that of control (F/F) and *Bis*-iCKO (iCKO) mice of the same age that received the same treatment starting on the same day (n = 4 for all three groups, all males). (A) Genotyping was performed using PCR to confirm Cre expression in iCKO and α-MHC-Cre

mice. The primers used for Cre and GAPDH were as follows. Cre, 5'-GTTTCACTGGTTATGCGGCGG (forward) and 5'-TTCCAGGGCGCGAGTTGATAG (reverse); GAPDH, 5'-GGTGTGAACGGATTTGGCCGTATT (forward) and 5'- GGCCTTGACTGTGCCGTTGAATTT (reverse). (B) The ratio of heart weight to body weight for each of the three groups indicates that the values for the Cre mice did not increase as much as those for the *Bis*-iCKO mice. (C) Echocardiographic tracing of the three groups before (D0) and after (D28) tamoxifen injection. (D) LVEF-delta and FS-delta changes on D28 also demonstrate that the LV function in Cre mice was not significantly impaired. (E) Expression profiles of BIS, ANF, β-MHC, Col1a1, and Col3a1 mRNA were determined using qRT-PCR. The heart failure markers and profibrotic mRNAs in Cre mice exhibited similar levels as the values for the F/F groups, whereas *Bis*-iCKO mice exhibited serious cardiac dysfunction, as shown in Fig. 2. (F) Western blotting assay results also show that BIS and HSPB expression was not reduced in the Cre groups. The data are presented as the mean ± SD from three or four mice. *p <0.05; **p <0.01; ***p <0.001 (compared with the values from F/F mice). ns, not statistically significant.
